# Cognitive Processes and Personality Traits Underlying Four Phenotypes of Susceptibility to (Mis)Information

**DOI:** 10.3389/fpsyt.2022.912397

**Published:** 2022-06-15

**Authors:** Michal Piksa, Karolina Noworyta, Jan Piasecki, Pawel Gwiazdzinski, Aleksander B. Gundersen, Jonas Kunst, Rafal Rygula

**Affiliations:** ^1^Affective Cognitive Neuroscience Laboratory, Department of Pharmacology, Maj Institute of Pharmacology Polish Academy of Sciences, Kraków, Poland; ^2^Department of Philosophy and Bioethics, Faculty of Health Sciences, Jagiellonian University Medical College, Kraków, Poland; ^3^Department of Psychology, University of Oslo, Oslo, Norway

**Keywords:** cognitive, personality, psychological, phenotype, susceptibility, fake news, misinformation, anxiety

## Abstract

Misinformation on social media poses a serious threat to democracy, sociopolitical stability, and mental health. Thus, it is crucial to investigate the nature of cognitive mechanisms and personality traits that contribute to the assessment of news items' veracity, failures in the discernment of their truthfulness, and behavioral engagement with the news, especially if one wants to devise any intervention to stop the spread of misinformation in social media. The current research aimed to develop and test a 4-fold taxonomy classifying people into four distinct phenotypes of susceptibility to (mis)information. In doing so, it aimed to establish differences in cognitive and psychological profiles between these phenotypes. The investigated cognitive processes included sensitivity to feedback, belief updating, and cognitive judgment bias. Psychological traits of interest included the Big Five model, grandiose narcissism, anxiety, and dispositional optimism. The participants completed online surveys that consisted of a new scale designed to classify people into one of four phenotypes of susceptibility to (mis)information, advanced cognitive tests, and reliable psychological instruments. The four identified phenotypes, Doubters, Knowers, Duffers, and Consumers, showed that believing in misinformation does not imply denying the truth. In contrast, the numerically largest phenotypes encompassed individuals who were either susceptible (Consumers) or resistant (Doubters), in terms of veracity judgment and behavioral engagement, to any news, regardless of its truthfulness. Significantly less frequent were the phenotypes characterized by excellent and poor discernment of the news' truthfulness (the Knowers and the Duffers, respectively). The phenotypes significantly differed in sensitivity to positive and negative feedback, cognitive judgment bias, extraversion, conscientiousness, agreeableness, emotional stability, grandiose narcissism, anxiety, and dispositional optimism. The obtained results constitute a basis for a new and holistic approach in understanding susceptibility to (mis)information as a psycho-cognitive phenotype.

## Introduction

The problem of misinformation has received increasing research interest as events such as the 2016 United States presidential elections or the Brexit referendum in Great Britain drew attention to the power of fake news in influencing public opinion about highly consequential issues (1). Together with the COVID-19 outbreak, the beginning of 2020 has born wild conspiracy theories. For instance, several theories focused on Bill Gates, alleging that he created the virus himself, had patented the cure and was conspiring to use a coronavirus vaccine as a ploy to monitor people through an injected microchip or quantum-dot spy software (2). These false claims proliferated and gradually flooded the media and mainstream. Indeed, for the individuals and organizations involved in the spread of such misinformation, the pandemic became a gilded opportunity. They started capitalizing on both the many unknowns about the SARS-CoV-2 virus and the disease it causes, as well as many legitimate questions about the safety and efficacy of vaccines developed at unprecedented speed (2). Although now, almost 2 years later, we know much more about the origin and mechanism of this misinformation (3), our knowledge about the cognitive and psychological factors responsible for susceptibility to this kind of news still remains scarce. Thus, an important research challenge may be to determine how people assess the truthfulness of information and how those decisions may be associated with their psychological predispositions. Below, we present several diverse cognitive and psychological factors that may be important when investigating the individual differences in susceptibility to fake news in the context of social media, where individuals are not only passive recipients of information but can also actively engage with news items by liking and sharing them with other users.

Deciding whether to believe information involves several cognitive and motivational processes, including the ability to differentiate between truth and falsehood based on analytical and reflective reasoning, the ability to update beliefs in response to new information, sensitivity to positive and negative reinforcement, and optimistically/pessimistically biased judgment. The role of analytical and reflective reasoning in veracity judgment has been recently demonstrated experimentally by several studies. They show that people are able to override incorrect intuitions via analytical thinking (4–6) and those who do not reflect sufficiently on their prior knowledge often fail to discern truth from falsehood (7). Surprisingly, the role of the other abovementioned, affect-dependent cognitive processes (8), although intuitive, to the best of our knowledge has never been established experimentally. For example, a reduced ability to update beliefs in response to information that is concordant/discordant with people's partisanship may lead to a false valuation of certain news as true or false based on their cognitive utility (9) Similarly, affect-dependent changes in sensitivity to feedback and pessimistic/optimistic judgment bias could reduce the ability to correctly infer truthfulness based on the affective utility of the information.

Individual schemas of cognitive processes, along with emotional and behavioral patterns, constitute a more general concept of personality (10). Various personality traits have been postulated to be involved in the way we process information (11), yet there have been very few attempts to explain the role of personality differences in the susceptibility to misinformation (12). It is rather puzzling given that the Big Five personality traits, extraversion, conscientiousness, agreeableness, openness to experience, and neuroticism [the Five-Factor Model (13)], as well as anxiety, understood as a stable personality characteristic (14, 15), have the potential to shape humans' perception of truthfulness. Moreover, grandiose narcissism (16) and optimism (17) might influence behavioral engagement, e.g., information sharing. Indeed, agreeableness, conscientiousness, and openness to experience have already been proven to negatively correlate with the perceived reliability of political misinformation (18). A similar correlation was observed following a procedure of anxiety induction (19), which decreased the perceived reliability of false information. In turn, research on narcissism has provided some indirect evidence suggesting that “self-lovers” might be more susceptible to “alternative” facts (20). Given this broad spectrum of possible interrelations, it is surprising how limited our knowledge is of the links between personality traits and susceptibility to (mis)information.

The constant evaluation of information is a fundamental process of human cognition and is integral to learning, social engagement, and decision-making (9, 21, 22). As such, susceptibility to fake news should not be investigated in isolation but should be considered in the broader context of overall susceptibility to information, which can be operationalized as judgment of its veracity and behavioral interaction with it (e.g., liking, sharing on social media) (6, 23). Here, we propose a framework that is based on the simultaneous analysis of susceptibility to true and fake news and identification of four patterns of this susceptibility: (1) susceptible to any kind of information, regardless of its truthfulness, (2) susceptible only to true news and unsusceptible to fake news; (3) susceptible only to fake news and insensitive to the truth; and (4) susceptible to any kind of information, regardless of its truthfulness. These patterns could be operationalized as the following phenotypes: (1) Consumers; (2) Knowers; (3) Duffers, and (4) Doubters (**Figure 3**). Such a framework offers a structure for characterizing and quantifying individual differences in susceptibility to (mis)information and allows for a nuanced test of its underlying cognitive and psychological traits.

In the present study, using Prolific Academic linked with Qualtrics and Millisecond Inquisit web testing platforms, we recruited a sample of participants and assessed their suscpetibiliy to various true and false news regarding the COVID-19 pandemic. Based on this assessment, we classified each participant into one of the four phenotypes of information susceptibility: Consumers, Knowers, Duffers, and Doubters. The participants were further tested with regard to the abovementioned cognitive processes using experimental paradigms, such as the Ambiguous-Cue Interpretation test [ACI (24)], assessing cognitive judgment bias, the Probabilistic Reversal Learning test [PRL (25)], measuring sensitivity to positive and negative reinforcement and cognitive flexibility, and the Belief Updating Test [BUT (26)], measuring asymmetry in updating one's beliefs based on the type of information obtained. Participants were also tested with regard to their personality traits using questionnaires including the Life Orientation Test-Revised [LOT-R (17)] allowing measurement of dispositional optimism; the Ten-Item Personality Inventory [TIPI (27)] assessing the Big Five personality traits (extraversion, conscientiousness, agreeableness, openness to experience and neuroticism); the Trait Anxiety Scale [TAS (14)] measuring their level of dispositional anxiety; the Grandiose Narcissism Scale [GNS (28)] evaluating self-perceived authority, self-sufficiency, superiority, vanity, exhibitionism, entitlement, and exploitativeness; and the Sensitivity to Punishment and Sensitivity to Reward Questionnaire [SPSRQ-RC (29)] and the BIS/BAS (behavioral inhibition system/behavioral activation system) Scale (30), which were aimed at self-assessment of reinforcement sensitivity.

## Materials and Methods

### Participants

**Figure 1:**
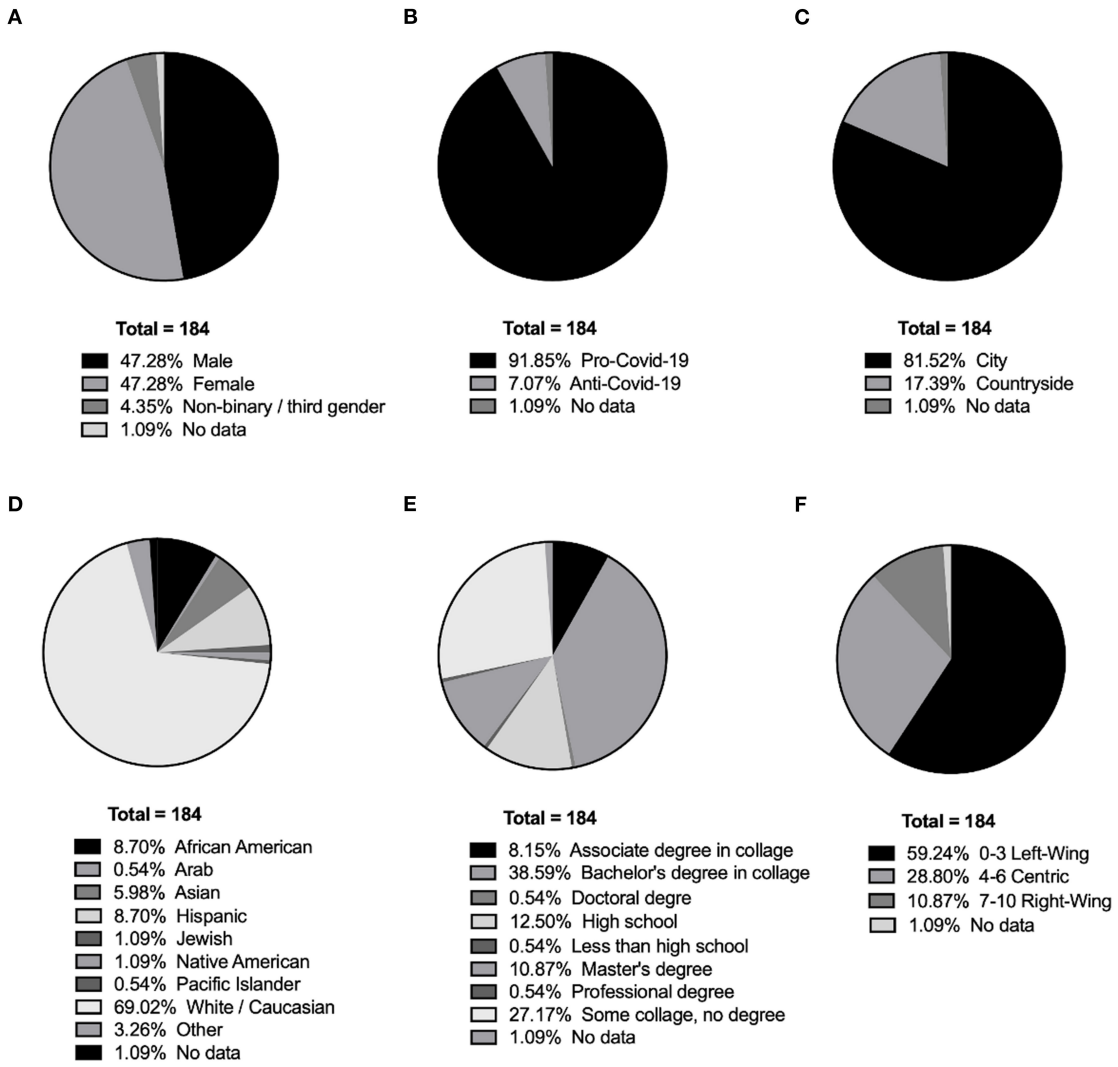
The demographics of the studied sample: **(A)** gender; **(B)** COVID-19 attitude; **(C)** place of living; **(D)** ethnicity; **(E)** highest completed education; **(F)** cumulative political orientation.

A power analysis using G^*^Power 3.1 (31) indicated that a total sample of 172 participants would give 90% power to detect medium effects (f = 0.5) in an analysis of differences between phenotypes of sensitivity to (mis)information using *t*-test, at an alpha of 0.05. Participants were recruited by Prolific Academic. During the Prolific prescreening stage, we excluded participants who had declared any hearing or vision difficulties or had no access to a computer, which was necessary for performing cognitive tests. To receive reliable answers, we recruited only the people who had previously participated in a minimum of 100 studies, with an acceptance rate of ≥95% for the submitted surveys. Additionally, we limited the number of previous participations to 500 to avoid people who had already conducted a high number of surveys. During the survey, the participants had to pass four attentional checks (“*It is important that you pay attention to this study. Please tick ‘Somewhat agree”*) and all participants answered all checks correctly. The targeted number of participants was 200 adult Americans; however, due to dropouts as a result of technical problems during the experiment (*n* = 16), we analyzed data for *N* = 184 (M_Age_ = 29.9, SD = 8.73). The final sample included 87 males, 87 females, and 8 non-binary people (Figure 1A). Two participants did not fill in the demographic data but were included in the analysis. The majority of participants (*n* = 169) declared that the COVID-19 pandemic is a danger (on a Likert scale 1–3), while the remaining 13 people declared that they believed the COVID-19 pandemic is a hoax (on a Likert scale 4–6, Figure 1B). The majority of people lived in a city (Figure 1C). The majority declared their ethnicity White/Caucasian, followed by African American, Hispanic, Asian, Jewish, Native American, Arab, Pacific Islander, and other (see Figure 1D). The highest level of completed education was a bachelor's degree, followed by some college but no degree, high school, master's degree, associate degree, doctoral degree, less than high school, and professional degree (Figure 1E). The participants were also asked about their political orientation—the majority of the sample (Figure 1F) had a left-wing orientation (on a scale from 0—left-wing to 10—right-wing, scoring 0 to 3), followed by centric (scoring from 4 to 6), and right-wing (scoring from 7 to 10).

### Susceptibility to (Mis)Information Scale

**Figure 2:**
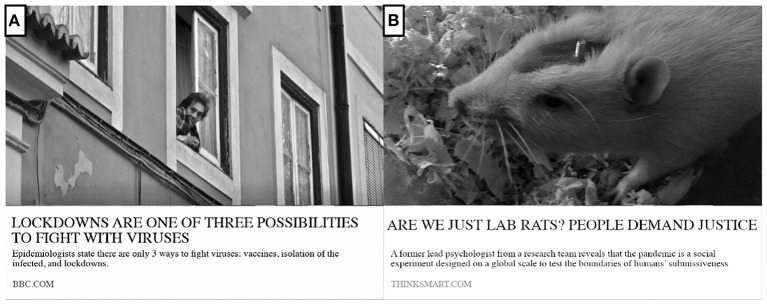
Examples of news headlines used in the current research to evaluate individuals' susceptibility to (mis)information **(A)** true news; **(B)** fake news.

The susceptibility to (mis)information was measured using a newly created scale based on 24 news headlines in a Facebook-like format. The topic of the news was connected to the COVID-19 pandemic. Half of the news presented true information, which was obtained from research reports and official World Health Organization guidelines and statistics (32). The other half presented fake information, which was created by the research team. Examples of true news and fake news are shown in Figure 2, and the whole scale can be accessed at (33).

Susceptibility to information was defined on two levels. The participants were asked to evaluate each item on the scale in terms of its veracity (“*Do you think the news above is true?”*, where 1 was “*definitely false*” and 6 was “*definitely true*”), and the probability of engagement with it (willingness to like (“*On social media, I would give a ‘like’ to this news”*, where 1 was “*totally disagree*” and 6 was “*totally agree*”), and willingness to share *(“I would share this news on my social media profile,”* where 1 was “totally disagree,” and 6 was “*totally agree*”). There was a positive correlation between willingness to share and willingness to like (for fake items: r = 0.88, *P* < 0.001; for true items: r = 0.91, *P* < 0.001).

By averaging the scores of all true news and fake news items, four variables emerged: true news veracity judgment (Cronbach's α = 0.73), fake news veracity judgment (Cronbach's α = 0.68), engagement with true news rating (Cronbach's α = 0.93), and engagement with fake news rating (Cronbach's α = 0.88).

### Phenotypes of Susceptibility to (Mis)Information

**Figure 3:**
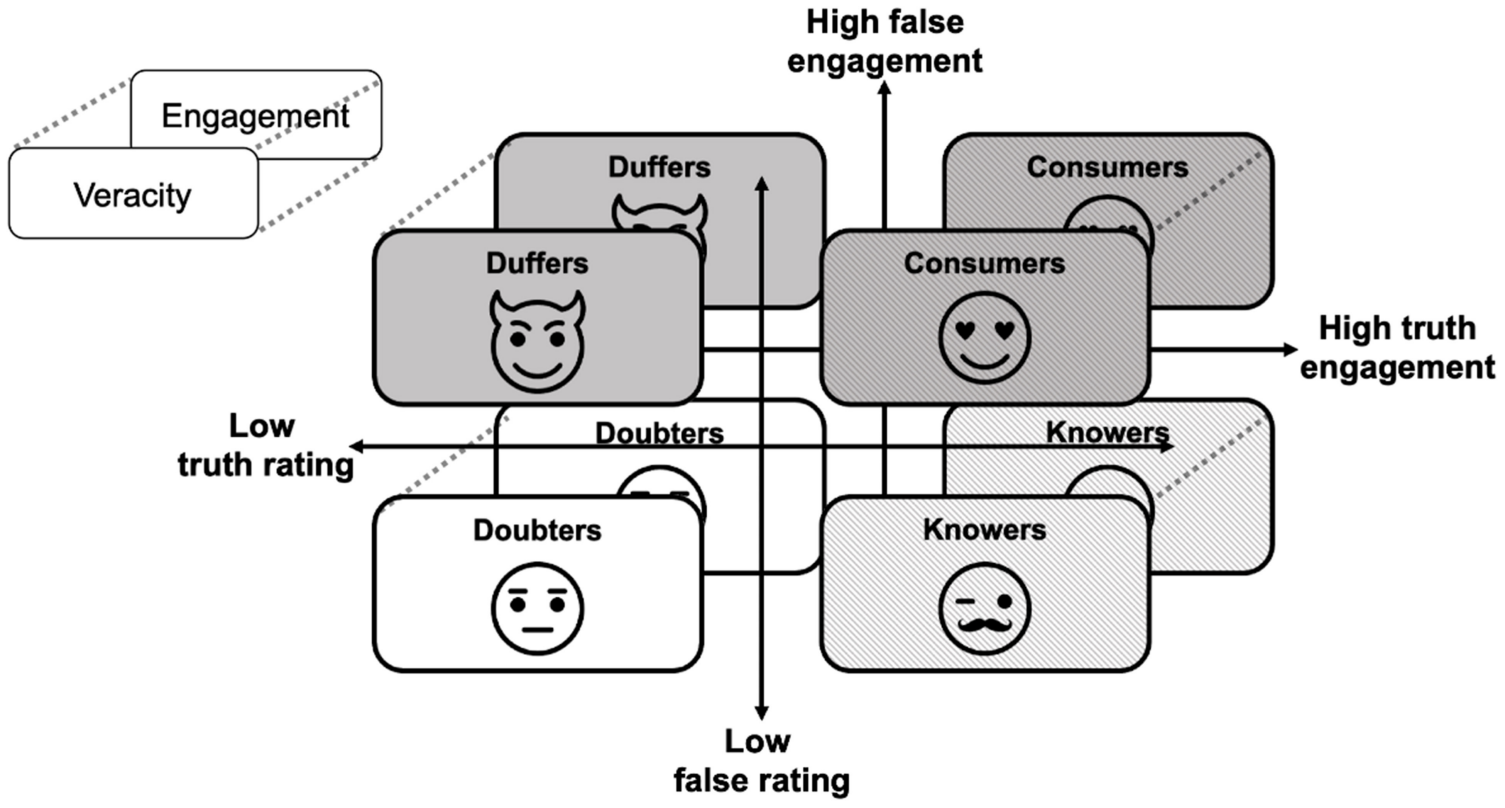
Four phenotypes of susceptibility to (mis)information. Consumers—the people highly rating veracity of and/or engagement with any kind of information; Knowers—the people highly rating veracity of and/or engagement with true news and low rating veracity of and/or engagement with fake news; Duffers—the people highly rating veracity of and/or engagement with fake news and low rating true news; Doubters—the people rating veracity of and/or engagement with all news low.

Based on the scores from SIS and a median split of the averaged true and averaged fake news veracity ratings, each participant was assigned to one of the four phenotypes of (mis)information susceptibility: Consumers (the people highly rating the veracity of any kind of information, regardless of its truthfulness), Knowers (the ones highly rating the veracity of true news and low rating the veracity of fake news), Duffers (opposite to the knowers: highly rating the veracity of fake news and low rating the veracity of true news), and Doubters (the people evaluating all news as untrustworthy). The same classification was conducted based on engagement with the news scores. Thus, this resulted in the differentiation of two separate types of phenotypic classification, one based on veracity and the other on engagement ratings (Figure 3).

### Cognitive Tests

#### Ambiguous Cue Interpretation

**Figure 4:**
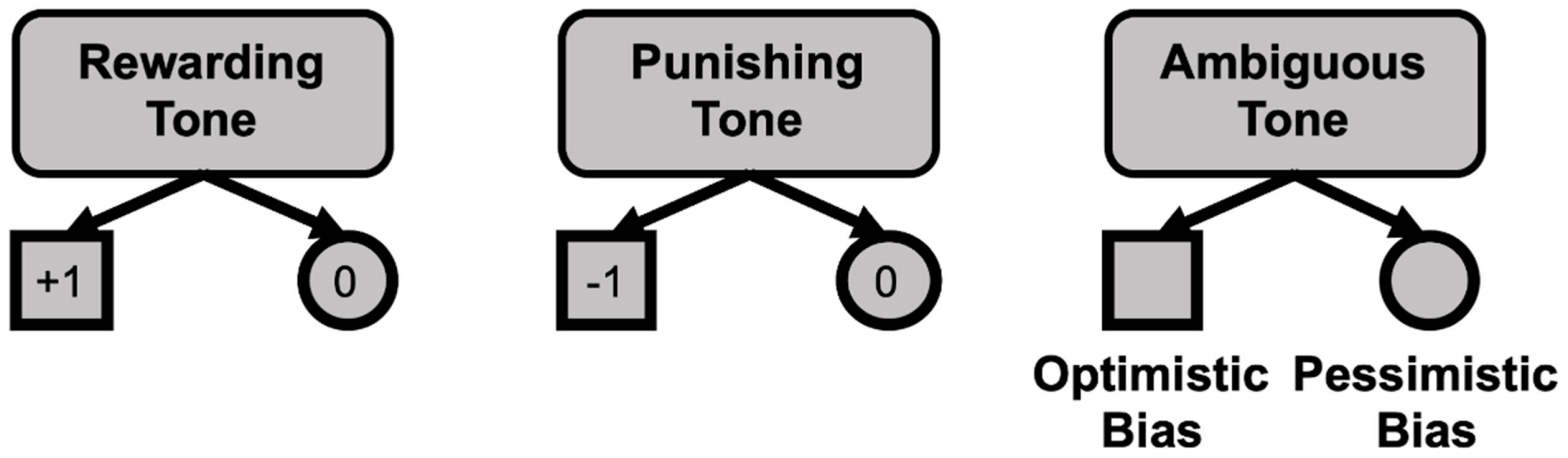
Schematic representation of the ambiguous interpretation test. Choosing the square following the rewarding tone gave 1 point; choosing the circle following the rewarding tone did not affect the score; choosing the square following the punishing tone took 1 point away; choosing the circle following the punishing tone allowed the respondent to avoid losing 1 point; choosing the square following the ambiguous tone indicated optimistic bias; and choosing the circle following the ambiguous tone indicated pessimistic bias.

To experimentally evaluate cognitive judgment bias (optimism/pessimism), the participants were tested using the ACI test. This procedure was adapted from Schick et al. (24) and modified for online testing. In this experimental paradigm, participants initially learned to discriminate two stimuli (tones of different frequencies of either 1,164 or 1,000 Hz), which acquired emotional and motivational value due to subsequent feedback (gaining points or avoidance of losing points). Following the tone predicting a reward, the participants had to press a symbol on the computer screen (square) to obtain one point. By the same token, following the tone predicting a punishment, the participants had to press another symbol (circle) to avoid losing one point. The tones were counterbalanced across the subjects. After this acquisition phase, the test phase introduced ambiguous stimulus (a tone of intermediate frequency-−1,078 Hz—to the tones predicting reward and punishment). The reaction to this tone (choosing the square or circle) served as a measure of judgment bias, indicating the participants' expectation of rewarding or potentially punishing effects of their decision (Figure 4). The testing phase consisted of 30 trials in total, 10 trials for each tone: rewarding, punishing, and ambiguous, presented in a pseudorandom order. During ACI testing, the responses to each tone (positive, ambiguous, and negative) were scored and analyzed as the proportion of the total number of responses to a given tone. To calculate the cognitive bias index, the proportion of negative responses to the ambiguous cues was subtracted from the proportion of positive responses, resulting in values ranging between −1 and +1, where values above 0 indicate an overall positive judgment and “optimistic” interpretation of the ambiguous cue.

#### Belief Updating Test

**Figure 5:**
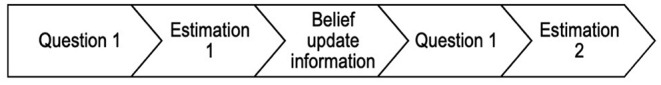
Schematic representation of the belief updating test.

To further test whether an optimistic bias could differ in phenotypes of susceptibility to (mis)information, participants completed a Belief Updating Test (BUT) (26). During the test, participants provided estimates of their likelihood of experiencing 10 different types of adverse life events (e.g., Alzheimer's disease, robbery). After each trial, they were presented with a pseudoactual probability of that event occurring to an average person in their environment. Subsequently, the ability of participants to use this information to update their predictions was assessed by asking them again for their estimates (Figure 5). The abovementioned pseudoactual probability was calculated by a simple equation y = 1.22x for half of the events and y = 0.78x for the other half (y—the feedback information, x—the first estimation). The first equation makes the feedback 22% higher than the estimated probability, whereas the second one lowers it by 22%. Unlike 20 or 25%, the chosen values lowered the risk that the participants would realize the mechanism behind the test. Additionally, this approach was used to avoid the complexity of estimating the actual probability of certain events under individual circumstances and to ensure that half of the presented probabilities were optimistic (for y = 0.78) and the other half was pessimistic (y = 1.22x). The belief updating scores were calculated using the following equation for each event:


$$\begin{array}{l} \text{O }=\;-1^{*}\frac{\text{E}2-\text{E}1}{\text{E}1-0.78\text{E}1}\\ \text{P }=\;-1^{*}\frac{\text{E}2-\text{E}1}{\text{E}1-1.22\text{E}1} \end{array}$$

where O is Optimistic belief updating, P is Pessimistic Belief Updating, E1 is First estimation, and E2 is Second estimation.

For O or *P* = 0, no belief update occurred. For O or *P* = 1, the belief update relied completely on the feedback. When O or *P* > 0 and < 1, the update partially relied on the feedback. When O or *P* > 1, the update exceeded the feedback, and when O or *P* < 0, the update was negatively modulated by the feedback. The final two scores Pessimistic Belief Updating Index and Optimistic Belief Updating Index were calculated as the means from the respective events.

#### Probabilistic Reversal Learning

To test how sensitivity to feedback differs in susceptibility to (mis)information, the participants completed the Probabilistic Reversal Learning (PRL) (25).

**Figure 6:**
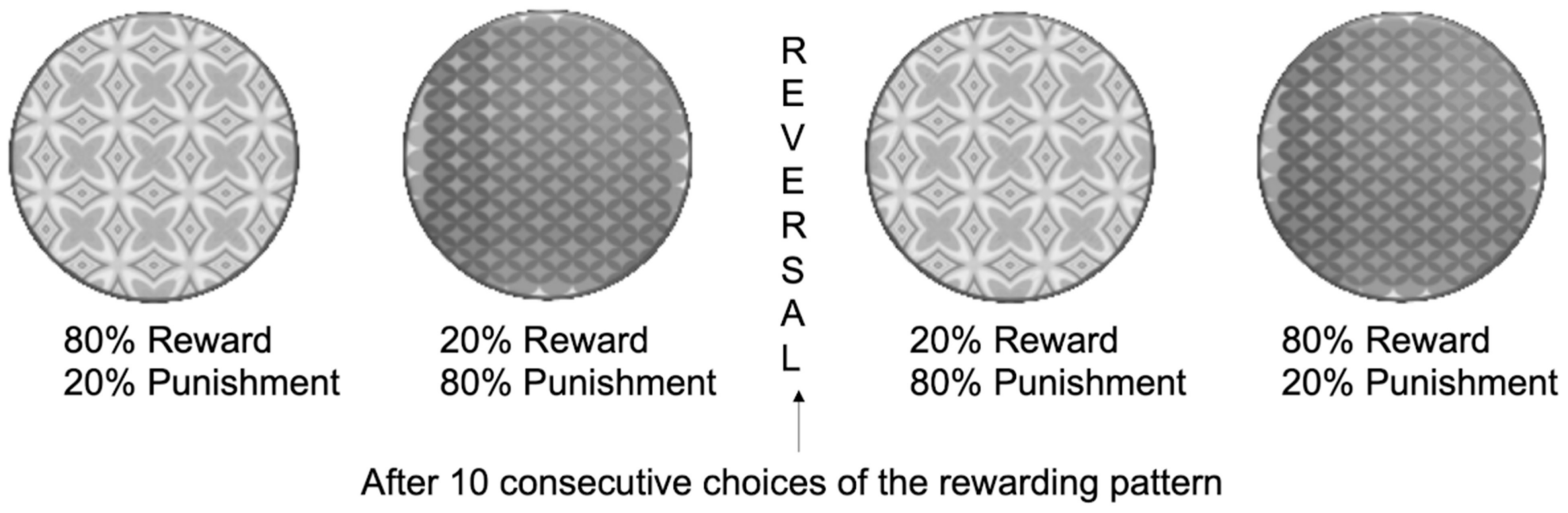
Schematic representation of the probabilistic reversal learning test with examples of presented stimuli.

In this task, for each trial, two stimuli (abstract, complex, colorful patterns on a computer screen, an example is presented in Figure 6) were presented simultaneously on the left and right sides of the screen (location randomized). Using trial-and-error feedback after each response, participants learned to select the stimulus that was usually correct (rewarded on 80% of trials and punished or unrewarded on 20% of trials) and to avoid the stimulus that was usually incorrect (punished or unrewarded on 80% of trials and rewarded on 20% of trials). Responses were made by pressing the “E” or “I” button on a computer keyboard. On each individual trial, the stimuli were presented for 2,000 ms within which the response had to be made (or else a “too late” message was presented). Rewards and punishments were symbolic, in the form of a green smiley face for correct responses or a red sad face for incorrect responses, appearing together with the words “correct” or “incorrect” onscreen after each choice. The rules intermittently reversed (after 10 consecutive choices of the usually rewarded patterns) such that the stimulus that was usually rewarded became usually punished and vice versa. Consequently, participants had to adjust their responses to gain the reward and avoid punishment. On a minority of trials (20%), false-negative and false-positive feedback was provided to correct and incorrect responses, respectively, the so-called “probabilistic errors.” Participants performed three successive blocks of the task, each lasting 5 min. The use of probabilistic reinforcement increased the complexity of the task in such a way that the information from any given choice was insufficient to guide future behavior, and participants must have engaged cognitive functions to track the reward and punishment history for both stimuli to determine the stimulus that is more beneficial overall. For successful completion of the task, participants had to learn to ignore infrequent and misleading negative and positive feedback that arose from the probabilistic nature of the discrimination.

Four types of events were analyzed using this task: (1) a correct response followed by positive feedback, (2) a correct response followed by negative feedback (probabilistic punishment), (3) an incorrect response followed by negative feedback, and (4) an incorrect response followed by positive feedback (probabilistic reward). These four types of events were then subjected to the Win-shift/Lose-shift analysis, where behavior was analyzed according to the outcome of each preceding trial to assess the sensitivity of participants to positive and negative feedback. Rewarded outcomes followed by a decision to shift the response (Win-shifts) were counted separately for the correct and incorrect responses and expressed as a ratio of all rewarded outcomes on a given stimulus. These Win–shifts ratios were used as a measure of sensitivity to either true (if the rewarding outcome followed the choice of the correct stimulus) or misleading (if the rewarding outcome followed the choice of the incorrect stimulus) positive feedback (the smaller the ratio was, the higher sensitivity to positive feedback). Conversely, Lose-shift ratios were calculated by dividing punishing outcomes after which the subject changed the choice by the total number of punished trials on a given stimulus. These Lose-shift ratios represented sensitivity to either true (when the punishing outcome followed the choice of the incorrect stimulus) or misleading (when the punishing outcome followed the choice of the correct stimulus) negative feedback (the higher the ratio was, the higher the sensitivity to negative feedback). Additionally, by consecutively choosing 10 correct patterns, the participants achieved reversal. The number of achieved reversals indicated cognitive flexibility, as it requires the participants to be able to notice and adapt to constantly changing rules of the test (Figure 6).

### Personality Questionnaires

#### Ten Item Personality Inventory

To evaluate the differences between the phenotypes in the Big Five personality traits: extraversion, emotional stability, conscientiousness, agreeableness, and openness to experience [Five-Factor Model (13)], the previously phenotyped participants completed the Ten Item Personality Inventory (TIPI) (27). In this questionnaire, each trait (e.g., extraversion – “*I see myself as extraverted, enthusiastic*”) is defined by a mean score of answers to two questions given on a 7-point Likert scale [1—“*disagree strongly,”* 7—“*agree strongly”*; (one item from each pair is reverse-coded]. The mean scores for each trait and correlation values between the items from each pair were for extraversion: 3.10 ± 1.63; r = 0.62, *P* < 0.001, for emotional stability 4.21 ± 1.53; r = 0.50, *P* < 0.001, for conscientiousness: 4.89 ± 1.57; r = 0.58, *P* < 0.001, for agreeableness: 4.38 ± 1.16; r = 0.24, *P* < 0.001, and for openness: 5.08 ± 1.27; r = 0.29, *P* < 0.001.

#### Trait Anxiety Scale

To test the differences between the phenotypes in trait anxiety, the subjects were evaluated using the Trait Anxiety Scale (TAS) (14), adapted from its original Polish version (*pl. Skala Leku-Cecha, SL-C*). It is a 15-item questionnaire (15 items, e.g., “*I am afraid of failure*”; mean = 27.72 ± 8.81; Cronbach's α = 0.90; 4-point scale: “0—*never,” “1—seldom,” “2—sometimes,” “3—often”*) constructed to evaluate individuals' anxiety as a constant personality trait, which is defined as a tendency to perceive situations as dangerous or to expect future events to be threatening, which manifests by characteristic cognitive, affective, behavioral and somatic symptoms.

#### Grandiose Narcissism Scale

To measure the differences between the phenotypes in grandiose narcissism, the participants completed the Grandiose Narcissism Scale (GNS) (28). The scale consists of 33 questions (mean = 102 ± 22.87; Cronbach's α = 0.93), divided into 7 subscales: authority (e.g., “*I like to be in charge of things*”; mean = 16.81 ± 6.57; Cronbach's α = 0.94) as a preference to be in charge, self-sufficiency (e.g., “*I don't rely on other people to get things done*”; mean = 21.61 ± 4.08; Cronbach's α = 0.78) as a preference to work on one's own, superiority (e.g., “*I'm more talented than most other people*”; mean = 11.79 ± 4.32; Cronbach's α = 0.87) as thinking to be better than others, vanity (e.g., “*I care about how good I look*”; mean = 18.90 ± 5.37; Cronbach's α = 0.91) as paying attention to one's physical appearance, exhibitionism (e.g., “*I do things that grab people's attention*”; mean = 12.31 ± 4.81; Cronbach's α = 0.86) as a need to attract others' attention, entitlement (e.g., “*I expect to be treated better than average*”; mean = 10.90 ± 3.96; Cronbach's α = 0.81) as a desire of special treatment, and exploitativeness (e.g., “*I can be pretty manipulative*”; mean = 10.52 ± 4.78; Cronbach's α = 0.9) as a tendency to use others for personal gains. The items were presented in random order. The answers were given on a 6-point Likert scale, where 1 was “*strongly disagree,”* and 6 was “*strongly agree.”*

#### Life Orientation Test—Revised

To test whether phenotypes of susceptibility to (mis)information differ in dispositional optimism, the participants completed the Life Orientation Test—Revised (LOT-R) (17). This brief questionnaire consists of 10 items (e.g., “*In uncertain times, I usually expect best*”; mean = 11.84 ± 5.08; Cronbach's α = 0.87) with possible answers given on a 5-point Likert scale, where 1 was “*strongly disagree”* and 5 -was “*strongly agree.”* Four of the items are so-called ‘filters’ (used to mask the real purpose of the questionnaire), which are not included in the final score. The score of this scale can be interpreted as dispositional optimism—a personality trait that makes people have favorable expectations about future events. The LOT-R is a self-assessment questionnaire measuring personal opinion in contrast to the ACI, which tests reactions to specific stimuli.

#### Sensitivity to Punishment Sensitivity to Reward Questionnaire-Revision and Clarification

To evaluate the differences between phenotypes in self-assessed sensitivity to punishment and reward, the participants completed the Sensitivity to Punishment Sensitivity to Reward Questionnaire-Revision and Clarification (SPSRQ-RC), described and validated in detail by Conner et al. (29). The SPSRQ-RC is a questionnaire that measures self-assessed sensitivity to reinforcement. It consists of 20 items, and the answers for each question are given on a 5-point Likert scale, where 1 is “*very untrue”* and 5 is “*very true”*). Summing up answers from items responding to sensitivity to reward (e.g., “*I do things for quick gains”*; mean = 24.82 ± 7.09; Cronbach's α = 0.81) and sensitivity to punishment (e.g., “*I am a shy person”; mean* = *32.72* ± 9.39; Cronbach's α = 0.9) gives general scores of these sensitivities.

#### Behavioral Inhibition System/Behavioral Activation System Scale

To further evaluate the differences, participants completed the Behavioral Inhibition System / Behavioral Activation System (BIS/BAS) Scale (30). BIS/BAS are the constructs from Gray's biopsychological theory of personality (34). BIS is a neural system that drives motivation to avoid punishment, novelty, and negative situations. BIS is responsible for negative emotions such as fear or anxiety, whereas BAS is a system that motivates participants to gain rewards, is goal-oriented, and is responsible for positive emotions. The BIS/BAS Scale is an empirical approach to measure individual differences in the level of sensitivity of the previously mentioned systems. It consists of 24 items (four are fillers), each with 4-point scale answers, where 1 means “*very untrue for me,”* and 4 means “*very true for me.”* The questionnaire consists of four different subscales that do not sum up to a single factor—BIS (e.g., “*I worry about making mistakes*”; mean = 22.00 ± 4.00; Cronbach's α = 0.81), BAS Drive (e.g., “*I go out of my way to get things done*”; mean = 10.10 ± 2.47; Cronbach's α = 0.77), BAS Reward Responsiveness (e.g., “*When I get something I want, I feel excited and energized*”; mean = 16.57 ± 2.53; Cronbach's α = 0.73), and BAS Fun Seeking (e.g., “*I crave excitement and new sensations*”; mean = 11.46 ± 2.33; Cronbach's α = 0.66).

### Statistical Analysis

The data were analyzed using SPSS (version 25.0, SPSS Inc., Chicago, IL, USA). The normality of the data was verified using the Shapiro–Wilk test. To validate the four phenotypes of susceptibility to (mis)information, the differences between the groups of participants classified as susceptible/unsusceptible to true and fake news were analyzed using a two-way analysis of variances (ANOVA) with the between-subject factors of susceptibility to true news (high vs. low) and susceptibility to fake news (high vs. low), separately for veracity judgment and engagement with the news. The differences between the phenotypes were analyzed by planned comparisons between a) Duffers and Knowers and b) Consumers and Doubters using *t*-tests, or where normality was violated, using U Mann–Whitney's test. The planned comparison was done because Duffers and Knowers represent the axis of truth discernment, whereas Consumers and Doubters represent a general susceptibility to (mis)information. The descriptive statistics of every analysis can be found in Supplementary Table 1.

### Procedure

**Figure 7:**
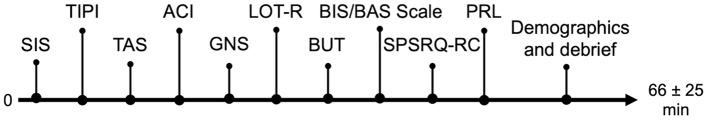
Schematic representation of the testing procedure. The tests were completed in the following order: SIS, Susceptibility to (mis)information scale; TIPI, Ten item personality inventory; TAS, Trait anxiety scale; ACI, Ambiguous cue interpretation test; GNS, Grandiose narcissism scale; LOT-R, Life orientation test revised; BUT, Belief updating test; BIS/BAS Scale, Behavioral Inhibition System/Behavioral Approach System scale; SPSRQ-RC, Sensitivity to punishment Sensitivity to reward questionnaire revision and clarification; PRL, Probabilistic reversal learning, and Demographic survey followed by a debrief.

The study was conducted between the 10th and 27th of August 2021. Eligible participants were recruited for the study via Prolific Academic, where they found essential information and instructions. Following informed consent, they were redirected to Qualtrics.com, where they completed the first part of the survey. In the second part of the study, they were asked to download the Millisecond Inquisit web application, where they completed the PRL test, and following completion of this task, they were redirected back to Qualtrics to fill in the demographic data and to be debriefed. The tests and questionnaires described in the previous paragraph were completed in the order presented in Figure 7. The mean time of the survey to be completed was 65.9 min with SD = 25.30. All participants were paid £ 9.38.

## Results

All data analyzed in this study have been made publicly available via Jagiellonian University Repository (33).

### Susceptibility to (Mis)Information Scale

#### Veracity Judgment Ratings

**Figure 8:**
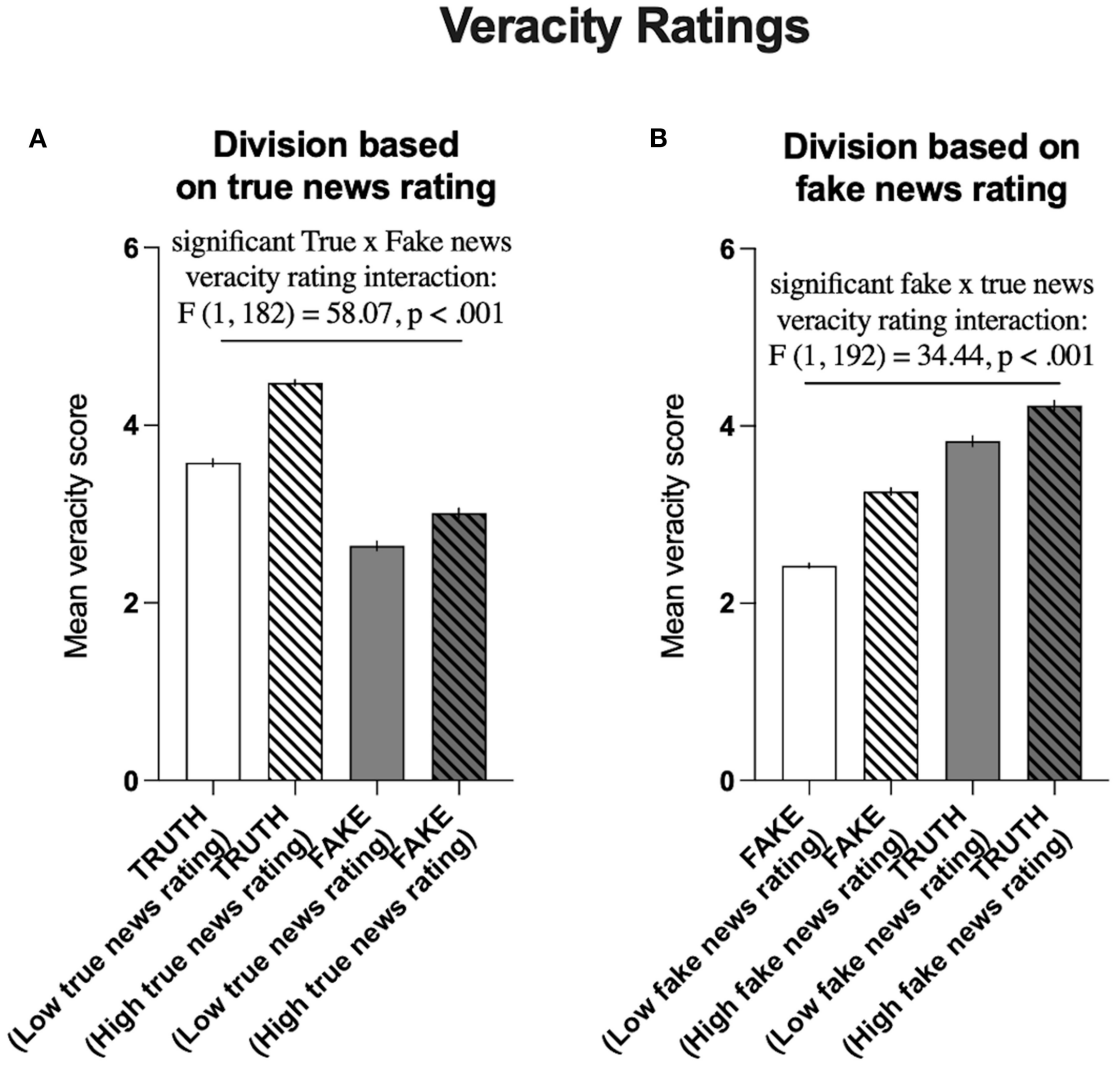
Division based on the average true and fake news ratings. **(A)** Comparison of the average true news veracity ratings (
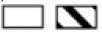
) with the average fake news veracity ratings (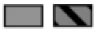) in groups that were differentiated based on the average low (*N* = 93) and high true (*N* = 91) news veracity ratings (
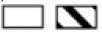
). **(B)** Comparison of the average fake news veracity ratings (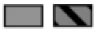) with the average true news veracity ratings (
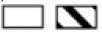
) in groups that were differentiated based on the average low (*N* = 96) and high (*N* = 88) fake news veracity ratings (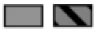). The data are presented as AVG veracity ratings ± SEM.

The median split of true news veracity ratings resulted in the differentiation of two groups of participants: those with scores above the median (high truth rating) and those with scores below the median (low truth rating). Comparison of the true news veracity ratings with fake news veracity ratings in these groups revealed that in general, the true news was rated higher in terms of veracity than the fake news [*F*_(1, 182)_ = 1,175, *P* < 0.001]. It also revealed a generalized effect of the veracity rating group, i.e., Participants who highly rated the true news also highly rated fake news [*F*_(1, 182)_ = 127.3, *P* < 0.001].Notably, the intergroup difference was significantly more pronounced for true news ratings than for fake news ratings [significant interaction: *F*_(1, 182)_ = 58.07, *P* < 0.001, Figure 8A].

Similarly, the median split of fake news veracity ratings resulted in the differentiation of another two groups of participants: those with scores above the median (high false rating) and those with scores below the median (low false rating). Comparison of the fake news veracity ratings with true news veracity ratings in these groups revealed that in general, the true news was rated higher in terms of veracity than the fake news [*F*_(1, 182)_ = 1,036, *P* < 0.001]. Moreover, it also revealed a generalized effect of news rating group, i.e., Participants who highly rated fake news also highly rated true news [*F*_(1, 182)_ = 177.8, *P* < 0.001]. Again, the intergroup difference was significantly more pronounced for fake news ratings than for true news ratings [significant interaction: *F*_(1, 192)_ = 34.44, *P* < 0.001, Figure 8B]. The significant statistical interactions between average scores of veracity in groups of highly/lowly rating true news and highly/lowly rating fake news validated the classification according to four phenotypes of (mis)information susceptibility based on veracity judgments.

#### Engagement Ratings

**Figure 9:**
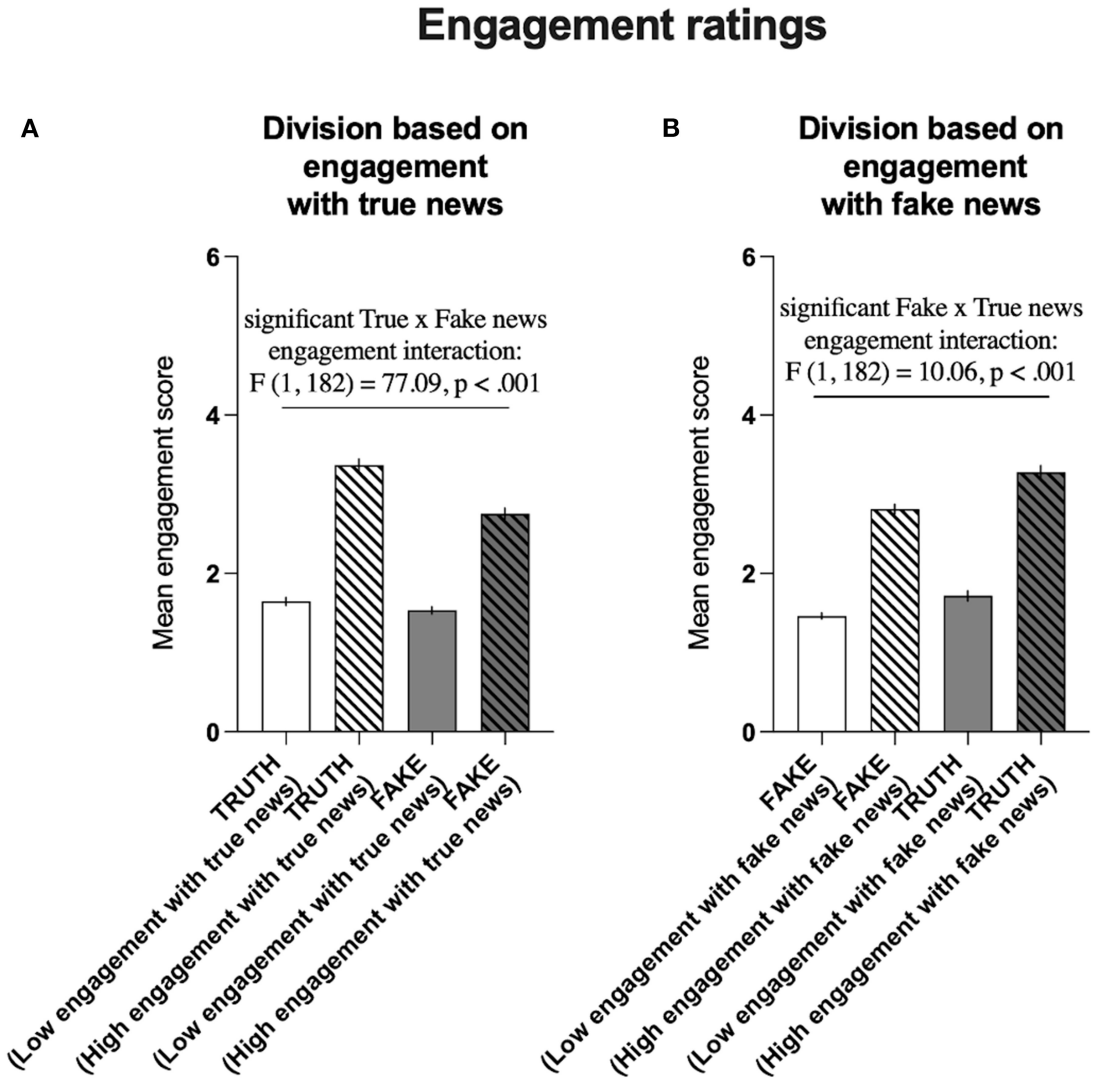
Division based on declared engagement with true and fake news. **(A)** Comparison of the declared average engagement with true news (
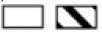
) with the average declared engagement with fake news (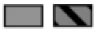) in groups that were differentiated based on the average low (*N* = 94) and high (*N* = 90) declared engagement with true news (
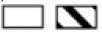
). **(B)** Comparison of the average declared engagement with fake news (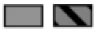) with the average declared engagement with true news (
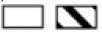
) in groups that were differentiated based on the average low (*N* = 93) and high (*N* = 91) engagement with fake news (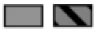). The data are presented as AVG engagement ratings ± SEMs.

Analogous to veracity ratings, the median split of the ratings of engagement with true news resulted in the differentiation of two groups of participants: those with scores above the median (highly engaged with true news) and those with scores below the median (unengaged with true news). Comparison of the ratings of engagement with fake news in these groups revealed that in general, the participants declared a higher rate of engagement with true news than with fake news [*F*_(1, 182)_ = 161.7, *P* < 0.001]. It also revealed a generalized effect of engagement with the news group, i.e., participants declaring high engagement with the true news were also declaring high engagement with the fake news [*F*_(1, 182)_ = 303.5, *P* < 0.001]. Notably, similar to the veracity ratings, the intergroup difference was significantly more pronounced for the declared engagement with true news than with the fake news (significant interaction: [*F*_(1, 182)_ = 77.09, *P* < 0.001, Figure 9A].

Similarly, the median split of the ratings of engagement with fake news resulted in the differentiation of two groups of participants: those with scores above the median (highly engaged with fake news) and those with scores below the median (unengaged with fake news). Comparison of the ratings of engagement with true news in these groups revealed that in general, the participants declared a higher rate of engagement with true news than with fake news [*F*_(1, 182)_ = 117.1, *P* < 0.001]. It also revealed a generalized effect of engagement with the news group, i.e., participants declaring high engagement with the fake news were also declaring high engagement with the true news [*F*_(1, 182)_ = 285.2, *P* < 0.001]. Again, similar to the veracity ratings, the intergroup difference was significantly more pronounced for the declared engagement with true news than with the fake news [significant interaction: *F*_(1, 182)_ = 10.06, *P* < 0.001, Figure 9B).

The significant statistical interactions between average scores of engagement in groups of high/lowly rating true news and high/lowly rating fake news validated the classification according to four phenotypes of (mis)information susceptibility based on engagement with the news.

### Frequency of Phenotypes of Susceptibility to (Mis)Information

Analysis of the phenotype distribution frequency in the investigated sample revealed that the most numerous were phenotypes of the Consumers (N_Veracity_ = 53, N_Engagement_ = 76) and the Doubters (N_Veracity_ = 58, N_Engagement_ = 79). Duffers and Knowers were significantly less numerous: Duffers (N_Veracity_ = 35, N_Engagement_ = 15) and Knowers (N_Veracity_ = 38, N_Engagement_ = 14).

### Differences Between Phenotypes of Veracity Rating

#### Cognitive Tests

##### PRL

The analysis of the Win-shift/Lose-shift data from PRL tests revealed a lack of significant interphenotypic differences in sensitivity to positive feedback between Doubters and Consumers (U = 1404, *P* = 0.43), between Knowers and Duffers (U = 490, *P* = 0.053), in negative feedback between Doubters and Consumers (U = 1,344, *P* = 0.26), between Knowers and Duffers (U = 593, *P* = 0.43) and in cognitive flexibility between Doubters and Consumers [*t*_(109)_ = 0.13, *P* = 0.90] or between Knowers and Duffers [*t*_(71)_ = 1.2, *P* = 0.23].

##### ACI

The analysis of choices following ambiguous cues in the ACI paradigm revealed a lack of significant interphenotypic differences in cognitive judgment bias between Doubters and Consumers (U = 1511, *P* = 0.88) and between Knowers and Duffers (U = 575.5, *P* = 0.32).

##### BUT

The analysis of optimistic and pessimistic belief updating indices revealed a lack of significant interphenotypic differences in optimistic belief updating (Doubters vs. Consumers, *t*_(107)_ = 0.99, *P* = 0.33; Knowers vs. Duffers, *t*_(71)_ = 0.14, *P* = 0.89) and in pessimistic belief updating (Doubters vs. Consumers, U = 1,334, *P* = 0.45; Knowers vs. Duffers, U = 604, *P* = 0.50).

#### Psychological Self-Assessment Questionnaires

##### TIPI

**Figure 10:**
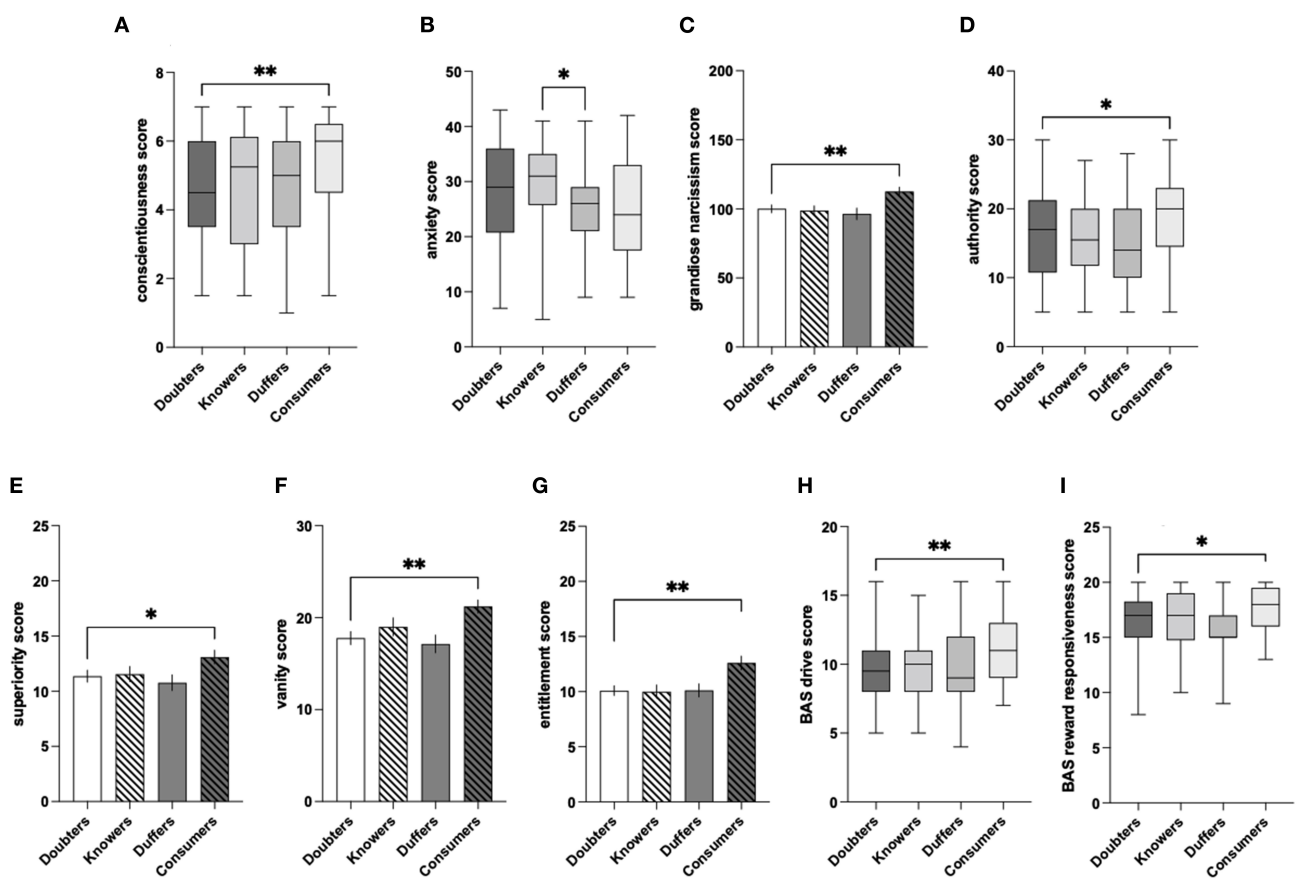
Interphenotypic differences in veracity ratings for **(A)** conscientiousness; **(B)** anxiety; **(C)** grandiose narcissism; **(D)** authority; **(E)** superiority; **(F)** vanity; **(G)** entitlement; **(H)** BAS Drive; and **(I)** BAS reward responsiveness. The data are presented as AVG ±SEM (the bar plots) or median with interquartile range (the box plots). *P ≤ 0.05 **P ≤ 0.01.

The analysis of the 5-factor model from TIPI revealed that Consumers were more conscientious than Doubters (U = 1083, *P* = 0.007; Figure 10A). There was no significant difference between Knowers and Duffers (U = 636.5, *P* = 0.76). Furthermore, no significant interphenotypic differences were revealed in extraversion (Doubters vs. Consumers, U = 1,351, *P* = 0.27; Knowers vs. Duffers, U = 628, *P* = 0.68), emotional stability (Doubters vs. Consumers, U = 1,265, *P* = 0.11; Knowers vs. Duffers, *t*_(71)_ = 0.07, *P* = 0.95), agreeableness (Doubters vs. Consumers, U = 1,531, *P* = 0.97; Knowers vs. Duffers, *t*_(71)_ = 1.172, *P* = 0.25) or openness to experience (Doubters vs. Consumers, U = 1,393, *P* = 0.391; Knowers vs. Duffers, U = 567.5, *P* = 0.28).

##### TAS

The TAS analysis revealed that Duffers were less anxious than Knowers (U = 444.5, *P* = 0.01; Figure 10B). There was no significant difference between Doubters and Consumers [*t*_(109)_ = 1.123, *P* = 0.26].

##### GNS

The analysis of GNS revealed that Consumers had higher scores than Doubters in grandiose narcissism [*t*_(109)_ = 3.03, *P* = 0.003; Figure 10C], authority (U = 1,193, *P* = 0.04; Figure 10D), superiority [*t*_(109)_ = 2.13, *P* = 0.04; Figure 10E], vanity [*t*_(109)_ = 3.38, *P* = 0.001; Figure 10F], and entitlement [*t*_(109)_ = 3.35, *P* = 0.001; Figure 10G]. There were no significant differences between Consumers and the Doubters in self-sufficiency (U = 1,359, *P* = 0.29), exploitativeness (U = 1,374, *P* = 0.34) or exhibitionism (U = 1,423, *P* = 0.50). The analysis revealed no significant differences between Knowers and Duffers in grandiose narcissism [*t*_(71)_ = 0.47, *P* = 0.65], authority [*t*_(71)_ = 0.37, *P* = 0.71], superiority [*t*_(71)_ = 0.79, *P* = 0.43], vanity [*t*_(71)_ = 1.35, *P* = 0.18], entitlement [*t*_(71)_ = 0.14, *P* = 0.89], self-sufficiency [*t*_(71)_ =1.25, *P* = 0.22], exploitativeness (U = 575.5, *P* = 0.32), and exhibitionism [*t*_(71)_ = 0.41, *P* = 0.68].

##### LOT-R

The analysis of LOT-R revealed no significant differences in dispositional optimism between Doubters and Consumers (U = 1,266, *P* = 0.11) or Knowers and Duffers (U = 624.5, *P* = 0.66).

##### BIS/BAS Scale

The analysis of variables from the BIS/BAS scale revealed that Consumers scored significantly higher than Doubters in BAS drive (U = 1,012, *P* = 0.002; Figure 10H) and BAS reward responsiveness (U = 1,152, *P* = 0.02; Figure 10I). There were no significant differences between these two phenotypes in BAS fun seeking [*t*_(109)_ = 1.83, *P* = 0.07] or BIS (U = 1,329, *P* = 0.22). Furthermore, there were no significant differences between Knowers and Duffers in BAS Drive [*t*_(71)_ = 0.12, *P* = 0.90], BAS reward responsiveness (U = 538.5, *P* = 0.16), BAS fun seeking [*t*_(71)_ = 1.25, *P* = 0.22] and BIS (U = 501, *P* = 0.07).

##### SPSRQ-RC

The analysis of SPSRQ-RC revealed a lack of significant interphenotypic differences in sensitivity to reward (Consumers vs. Doubters, *t*_(109)_ = 1.46, *P* = 0.15; Knowers vs. Duffers, *t*_(71)_ = 0.82, *P* = 0.42) and in sensitivity to punishment [Consumers vs. Doubters, U = 1,337, *P* = 0.24; Knowers vs. Duffers, *t*_(71)_ = 0.63, *P* = 0.53].

### Differences Between Phenotypes of Engagement Rating

#### Cognitive Tests

##### PRL

**Figure 11:**
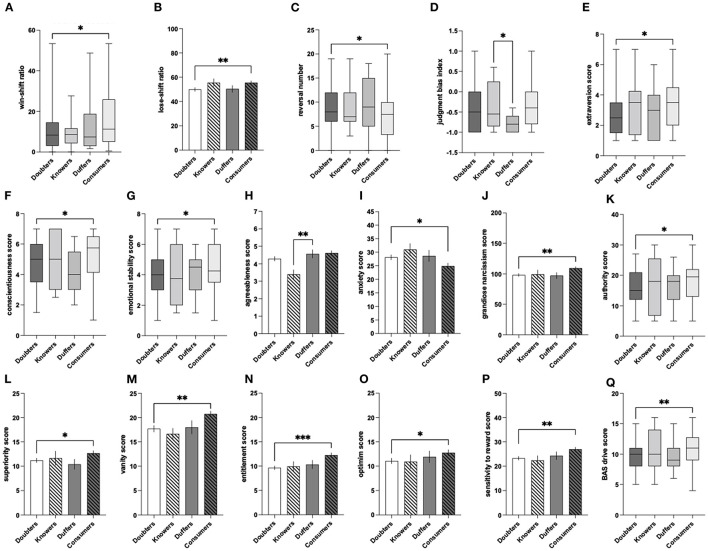
Interphenotypic differences in the ratings of declared engagement for **(A)** win-shift %; **(B)** lose-shift %; **(C)** reversals; **(D)** cognitive judgment bias; **(E)** extraversion; **(F)** conscientiousness; **(G)** emotional stability; **(H)** agreeableness; **(I)** anxiety; **(J)** grandiose narcissism; **(K)** authority; **(L)** superiority; **(M)** vanity; **(N)** entitlement; **(O)** optimism; **(P)** sensitivity to reward; **(Q)** BAS drive for **(A)** conscientiousness; **(B)** anxiety; **(C)** grandiose narcissism; **(D)** authority; **(E)** superiority; **(F)** vanity; **(G)** entitlement; **(H)** BAS drive; and **(I)** BAS reward responsiveness. The data are presented as AVG ± SEM (the bar plots) or median with interquartile range (the box plots). *P ≤ 0.05 **P ≤ 0.01 ***P ≤ 0.001.

The analysis of the win-shift/lose-shift data from PRL tests revealed that, compared to Doubters, Consumers were less sensitive to positive feedback (U = 2,439, *P* = 0.04; Figure 11A), more sensitive to negative feedback [*t*_(153)_ = 2,654, *P* = 0.009; Figure 11B], and less cognitively flexible, as indexed by fewer reversals (U = 2,373, *P* = 0.02; Figure 11C). Further analysis revealed a lack of significant differences between Knowers and Duffers in sensitivity to positive feedback (U = 104, *P* = 0.98), sensitivity to negative feedback [*t*_(27)_ = 1.199, *P* = 0.24], and cognitive flexibility [*t*_(27)_ = 0.32, *P* = 0.75].

##### ACI

Prescreening of the ACI data revealed the presence of 2 outliers (identified with the ROUT method), and these data were excluded from the analysis. In the ACI paradigm, Knowers more often identified the ambiguous tone as a cue predicting a reward than Duffers (U = 50, *P* = 0.04; Figure 11D). There was no significant difference between Consumers and Doubters (U = 2,817, *P* = 0.50).

##### BUT

The analysis of optimistic and pessimistic belief updating indices revealed a lack of significant interphenotypic differences between the groups of interest in optimistic belief updating (Consumers vs. Doubters (U = 2,734, *P* = 0.41); Knowers vs. Duffers (*t*_(27)_ = 0.42, *P* = 0.68)), and in pessimistic belief updating [Consumers vs.Doubters (U = 2,683, *P* = 0.45); Knowers vs. Duffers (U = 76, *P* = 0.51)].

#### Psychological Self-Assessment Questionnaires

##### TIPI

The analysis of the 5-factor model from TIPI revealed that, compared to Doubters, Consumers were more extraverted (U = 2,356, *P* = 0.02; Figure 11E), more conscientious (U = 2,411, *P* = 0.03; Figure 11F), and more emotionally stable (U = 2,449, *P* = 0.046, Figure 11G). There was no significant difference between Doubters and Consumers in agreeableness (U = 2,553, *P* = 0.10) and openness to experience (U = 2,950, *P* = 0.85).

In the case of agreeableness, Duffers scored higher than Knowers [*t*_(27)_ = 3.31, *P* = 0.003; Figure 11H]. However, the analysis revealed no significant differences between these phenotypes in extraversion [*t*_(27)_ = 0.44, *P* = 0.66], conscientiousness (U = 77.5, *P* = 0.24), emotional stability [*t*_(27)_ = 0.15, *P* = 0.87], and openness to experience (U = 68, *P* = 0.11).

##### TAS

The TAS analysis revealed that Doubters were more anxious than Consumers [*t*_(153)_ = 2.31, *P* = 0.02; Figure 11I]. There was no significant difference between Knowers and Duffers [*t*_(27)_ = 0.77, *P* = 0.45].

##### GNS

The analysis of GNS revealed that the Consumers had higher scores than Doubters in grandiose narcissism [*t*_(153)_ = 3.14, *P* = 0.002; Figure 11J], authority (U = 2,397, *P* = 0.03; Figure 11K), superiority [*t*_(153)_ = 2.18, *P* = 0.03; Figure 11L], vanity [*t*_(153)_ = 3.31, *P* = 0.001; Figure 11M], and entitlement [*t*_(153)_ = 4.23, *P* < 0.001; Figure 11N]. There were no significant differences between Doubters and Consumers in self-sufficiency (U = 2,717, *P* = 0.31), exploitativeness (U = 2,581, *P* = 0.13), or exhibitionism (U = 2,672, *P* = 0.24). The analysis revealed no significant differences between Knowers and Duffers in grandiose narcissism [*t*_(27)_ = 0.25, *P* = 0.81], authority [*t*_(27)_ = 0.54, *P* = 0.60], superiority [*t*_(27)_ = 0.71, *P* = 0.49], vanity [*t*_(27)_ = 0.75, *P* = 0.46], entitlement (U = 96.5, *P* = 0.72), self-sufficiency [*t*_(27)_ = 0.27, *P* = 0.79], exploitativeness (U = 103, *P* = 0.94), and exhibitionism [*t*_(27)_ = 0.25, *P* = 0.81].

##### LOT-R

The analysis of LOT-R revealed that Consumers were more optimistic than Doubters [*t*_(153)_ = 2.09, *P* = 0.040; Figure 11O]. No significant differences were found between Knowers and Duffers [*t*_(153)_ = 0.55, *P* = 0.59].

##### BIS/BAS Scale

The analysis of variables from the BIS/BAS scale revealed that Consumers scored significantly higher than Doubters in BAS drive (U = 2,102, *P* = 0.001; Figure 11Q). There were no significant differences between these two phenotypes in BAS fun seeking (U = 2,781, *P* = 0.43), BAS reward responsiveness (U = 2,681, *P* = 0.25), or BIS (U = 2,541, *P* = 0.10). Knowers and Duffers did not significantly differ in any of these parameters—BAS Drive [*t*_(27)_ = 1.01; *P* = 0.32], BAS fun seeking [*t*_(27)_ = 0.51, *P* = 0.62], BAS Reward Responsiveness (U = 99, *P* = 0.81), and BIS (U = 104, *P* = 0.97).

##### SPSRQ-RC

The analysis of SPSRQ-RC revealed that Consumers were more sensitive to reward than Doubters *t*_(153)_ = 3.25, *P* = 0.001; Figure 11P). There was no significant difference between these phenotypes in sensitivity to punishment (U = 2,682, *P* = 0.25). No significant differences were revealed between Knowers and Duffers in sensitivity to reward [*t*_(27)_ = 0.77, *P* = 0.45] and sensitivity to punishment [*t*_(27)_ = 1.1, *P* = 0.28].

## Discussion

The present study proposed the concept of four phenotypes of susceptibility to (mis)information that result from combining individual veracity ratings of true and fake news and similar classification based on behavioral engagement with true and fake news. Empirical implementation of this concept provides a holistic approach to the investigation of the susceptibility to (mis)information that had not previously been operationalized in the fake news research. The results of our study also revealed that phenotypes of susceptibility to (mis)information differed with respect to several cognitive processes and psychological traits. Both ways of phenotypic classification, which were established on the basis of the veracity ratings and the one established on the basis of engagement with the news ratings, revealed statistically significant interphenotypic differences in psychological traits, including conscientiousness, anxiety, narcissism, and BAS drive. The phenotypes based on engagement with the news differed from each other in extraversion, agreeableness, emotional stability, dispositional optimism, and sensitivity to reward. Moreover, they also differed in several cognitive processes, including sensitivity to positive and negative feedback measured in the PRL tests and cognitive judgment bias measured in the ACI paradigm.

When analyzing factors altering susceptibility to misinformation, it is important to consider not only the extent to which misinformation is believed in relation to true content (truth discernment) but also the overall degree to which information is believed, regardless of its truthfulness. This is important because although increasing or decreasing belief in true and false headlines to an equivalent extent does not affect truth discernment, it might still determine the effects of misinformation (7). To address this need, in our study, we introduced the innovative concept of four phenotypes of susceptibility to (mis)information that result from combining individual veracity ratings and/or engagement with true as well as false news. The resulting phenotypes of Doubters, Duffers, Knowers, and Consumers encompass four combinations of susceptibility to (mis)information allowing for a complex and holistic analysis of factors influencing susceptibility to misinformation itself and its position within the spectrum. The analysis of phenotypes' frequency distribution revealed that contrary to Duffers and Knowers, the most numerous phenotypes were Doubters and Consumers. While the higher frequency of phenotypic Doubters and Consumers vs. Knowers and Duffers, distinguished based on the engagement with information, seems intuitive—some people willingly share content and others rarely engage in any social media activity, the disproportion in the frequency of phenotypes distinguished based on veracity judgments was surprising. A possible explanation implies the “all-or-nothing” bias (35), which is a tendency to dichotomously perceive reality that drives people to choose extremes. In the case of the present study, the majority of the participants rated all news as true (Consumers) or all news as false (Doubters).

**Figure 12:**
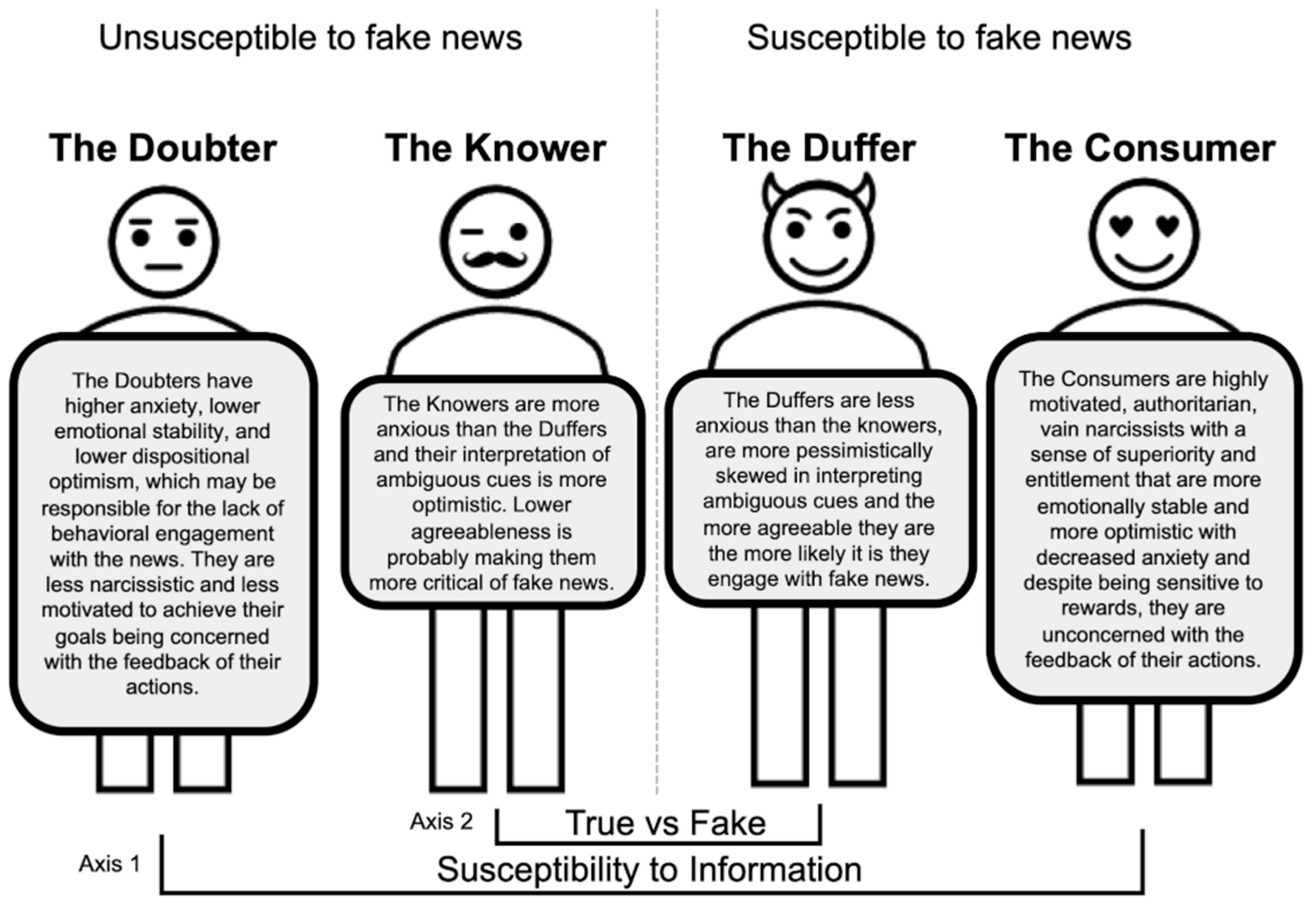
Psychological profiles of the phenotypes of susceptibility to (mis)information.

In the present study, the interphenotypic differences in cognitive and psychological traits were analyzed along two different axes: one encompassing a general susceptibility/unsusceptibility to information (Consumers vs. Doubters), and the other that differentiated people who were susceptible to fake news from those who were unsusceptible to this type of information (Knowers vs. Duffers). Importantly, they were analyzed not only on the level of basic veracity ratings but also in terms of behavioral engagement (liking, sharing). Performed analyses revealed (Figure 12) that the people highly rating the veracity of all incoming information (Consumers), compared to those who were less likely to believe any information (Doubters), could be described as highly motivated, authoritarian, vain narcissists with a sense of superiority and entitlement who are highly responsive to a reward. The higher levels of narcissism, vanity, sense of entitlement, and superiority also characterized Consumers classified in terms of behavioral engagement. People displaying this phenotype were more emotionally stable and optimistic than Doubters. Notably, high engagement with all sorts of news was characterized by decreased anxiety and volatility in using feedback to guide decisions about future actions, as indicated by higher levels of win-shift and lose-shift behaviors in the PRL task. The latter suggests that despite being sensitive to rewards, Consumers are unconcerned with the feedback of their actions. This might be explained by higher emotional stability and a lower level of dispositional anxiety, accompanied by optimism, which prevent Consumers from experiencing negative emotions connected to unflattering opinions of others, for example, on social media. In contrast, higher anxiety, lower emotional stability, and lower dispositional optimism may be responsible for the lack of behavioral engagement with the news by Doubters. It is worth noting that Consumers, in terms of both veracity rating and engagement with the news, also demonstrated significantly higher conscientiousness than Doubters. This observation suggests that Consumers might require more time and evidence to classify news as false and prefer to engage with any sort of information for further exploration.

Analysis of the second axis of vulnerability to the news, e.g., susceptibility to true vs. fake news, revealed that Duffers, distinguished based on their veracity judgments, were less anxious than Knowers. Two possible explanations might be considered. From one perspective, the existence of the pandemic is threatening, and people who deny the true information about it do so because they do not perceive it as dangerous enough to believe it. Another perspective suggests that Knowers who accept the danger of the pandemic are more anxious because of the threat behind it, whereas Duffers might find fake news anxiolytic. Whether low anxiety causes believing in false information or false information lowers anxiety is difficult to determine based on the results of this study, and the discussed causation should be further explored in future experiments.

The exploration of differences between Knowers and Duffers distinguished based on engagement with true and false information revealed that Duffers were more pessimistically skewed in interpreting ambiguous cues. This observation could explain why these people prefer to share and like false information, which often presents conspiracies that generally accuse some third persons or institutions to have malicious intentions. Engagement with fake news seems to escalate together with increased agreeableness. Indeed, while in the information bubble, persons who are more agreeable might be more likely to engage with fake news than others.

The empirical results reported herein should be considered in light of some limitations. Since social media constitute the natural environment where individuals interact with (mis)information, we designed our scale of susceptibility to (mis)information in a way that mimicked Facebook headlines. This approach, although broadly used in similar research, could be improved by conducting more ecological, real-time studies using algorithms tracking the behavior of users on social media platforms. This would help to eliminate situations where declared willingness to share or like given information in a survey might differ from actual sharing and liking behavior in social media. The second possible limitation might concern the online data collection and lack of control over the setting in which participants provided their responses, e.g., the PRL or ACI tests have never been previously used in the studies conducted online. This, however, has been mitigated by recruiting experienced but not professional individuals (see Methods) and performing attentional checks, which warranted motivation and devotion of the study participants.

## Conclusion

Our findings indicated the presence of various phenotypes of susceptibility to (mis)information, characterized by different clusters of cognitive and psychological traits. They also indicated that the concept of vulnerability to fake news cannot be investigated in isolation from the general susceptibility to information regardless of its veracity. Outlining the four phenotypes of susceptibility to (mis)information creates foundations for further research that should focus on the real-time behavior of people using social media and on the diagnosis of vulnerability to misinformation.

## Data Availability Statement

All data analyzed in this study have been made publicly available via Jagiellonian University Repository (33), and can be accessed here: https://ruj.uj.edu.pl/xmlui/handle/item/289808?locale-attribute=en.

## Ethics Statement

The studies involving human participants were reviewed and approved by the Bioethics Committee of Jagiellonian University in Krakow, Poland (1072.6120.66.2021 from 19 May 2021). The patients/participants provided their written informed consent to participate in this study.

## Author Contributions

JP, JK, and RR obtained funding. MP, KN, AG, JP, JK, and RR designed and planned the research. MP conducted the experiment and analyzed the data. MP and RR wrote the manuscript. MP, KN, JP, PG, AG, JK, and RR revised the the manuscript and contributed to the final discussion. All authors contributed to the article and approved the submitted version.

## Funding

The research leading to these results has received funding from the EEA Financial Mechanism 2014-2021. Project: 2019/35/J/HS6/03498.

## Conflict of Interest

The authors declare that the research was conducted in the absence of any commercial or financial relationships that could be construed as a potential conflict of interest.

## Publisher's Note

All claims expressed in this article are solely those of the authors and do not necessarily represent those of their affiliated organizations, or those of the publisher, the editors and the reviewers. Any product that may be evaluated in this article, or claim that may be made by its manufacturer, is not guaranteed or endorsed by the publisher.
