# Supplementary material for: Cognitive Processes and Personality Traits Underlying Four Phenotypes of Susceptibility to (Mis)Information
Source: Front Psychiatry. 2022 Jun 15;13:912397. doi: 10.3389/fpsyt.2022.912397 (PMC9240766; doi:10.3389/fpsyt.2022.912397)
Supplement: Supplementary file 1 [file Table_1.DOCX]

**Supplementary Materials**

Supplement1A)

Descriptive statistics of dependent variables in the phenotypes distinguished on a basis of veracity judgment.

Extra. – extraversion, Consc. – conscientiousness, Em. St. – emotional stability, Agree. – agreeableness, Open. – openness to experience, Author. – authority, Super. – superiority, Entitl. – entitlement, Self S. – self-sufficiency, Exploit. – exploitativeness, Exhib. – exhibitionism, Narcis. – grandiose narcissism, BAS D. – BAS drive, BAS F. – BAS fun seeking, BAS R. – BAS reward responsiveness, Optim. – optimism, Anxi – anxiety, SP – sensitivity to punishment, SR – sensitivity to reward, J. Bias – judgment bias, BUT O – optimistic belief updating, BUT P – pessimistic belief updating, Win – win-shift, lose – lose-shift, Rever. – reversals

Supplement 1B)

Descriptive statistics of dependent variables in the phenotypes distinguished on a basis of engagement with the news.

|  | The Doubters | | | | The Knowers | | | | The Duffers | | | | The Consumers | | | |
| --- | --- | --- | --- | --- | --- | --- | --- | --- | --- | --- | --- | --- | --- | --- | --- | --- |
|  | M | SD | Mdn | Mdn 95% CI | M | SD | Mdn | Mdn 95% CI | M | SD | Mdn | Mdn 95% CI | M | SD | Mdn | Mdn 95% CI |
| Extra. | 2.82 | 1.62 | 2.5 | 2, 3 | 3.32 | 1.89 | 3.5 | 1, 5 | 3.03 | 1.63 | 3 | 1, 4 | 3.35 | 1.58 | 3.5 | 2.5, 4 |
| Consc. | 4.73 | 1.54 | 5 | 4, 5.5 | 4.93 | 1.89 | 5 | 3, 7 | 4.07 | 1.36 | 4 | 3, 5.5 | 5.21 | 1.52 | 5.75 | 5, 6 |
| Em. St. | 3.98 | 1.37 | 4 | 3.5, 4 | 3.96 | 2.02 | 3.75 | 2, 6 | 4.07 | 1.52 | 4.5 | 3, 5 | 4.51 | 1.59 | 4.25 | 3.5, 5 |
| Agree. | 4.29 | 1.15 | 4 | 4, 4.5 | 3.39 | 0.98 | 3.5 | 2.5, 4.5 | 4.57 | 1.16 | 4.5 | 4, 5.5 | 4.61 | 1.16 | 4.5 | 4, 5 |
| Open. | 5.04 | 1.39 | 5.5 | 4.5, 6 | 4.93 | 1.11 | 4.5 | 4, 6.5 | 5.57 | 1.03 | 6 | 4.5, 6.5 | 5.05 | 1.19 | 5 | 4.5, 5.5 |
| Author. | 15.7 | 6.28 | 15 | 14, 18 | 17.36 | 8.76 | 18 | 6, 27 | 15.8 | 6.77 | 18 | 12, 20 | 18.08 | 6.26 | 19.5 | 17, 20 |
| Super. | 11.22 | 4 | 11 | 10, 12 | 11.64 | 5.41 | 13.5 | 5, 16 | 10.4 | 4.01 | 10 | 7, 14 | 12.68 | 4.4 | 14 | 11, 14 |
| Vanity | 17.71 | 6.07 | 18 | 13, 20 | 16.64 | 4.31 | 17.5 | 13, 20 | 18 | 5.33 | 19 | 13, 20 | 20.72 | 5.24 | 20 | 19, 22 |
| Entitl. | 9.65 | 3.49 | 10 | 9, 11 | 9.223 | 3.77 | 9 | 5, 14 | 10.33 | 3.35 | 9 | 8, 11 | 12.25 | 4.17 | 12 | 11, 14 |
| Self S. | 21.78 | 4.27 | 22 | 21, 24 | 21.86 | 4.61 | 21 | 19, 26 | 21.4 | 4.45 | 20 | 17, 26 | 21.43 | 3.79 | 21 | 21, 22 |
| Exploit. | 9.95 | 4.61 | 9 | 7,10 | 10.71 | 5.57 | 9 | 6, 16 | 10.47 | 4.82 | 10 | 5, 15 | 11.09 | 4.83 | 10.5 | 9, 13 |
| Exhib. | 12.13 | 5.07 | 12 | 10, 14 | 11.07 | 4.16 | 11.5 | 7, 14 | 10.67 | 4.64 | 10 | 6, 15 | 13.05 | 4.63 | 13 | 11, 15 |
| Narcis. | 98.13 | 22.24 | 100 | 93, 105 | 99.21 | 26.74 | 104 | 76, 128 | 97.07 | 20.02 | 96 | 84, 119 | 109.3 | 22.09 | 108.5 | 104, 116 |
| BAS D. | 9.51 | 2.15 | 10 | 9, 10 | 10.57 | 3.44 | 10 | 8, 14 | 9.47 | 2.36 | 9 | 8, 11 | 10.75 | 2.47 | 11 | 10, 12 |
| BAS F. | 11.33 | 2.42 | 11 | 11, 12 | 11.93 | 2.06 | 12 | 9, 14 | 11.53 | 2.13 | 11 | 11, 13 | 11.49 | 2.34 | 12 | 11, 13 |
| BAS R. | 16.24 | 2.68 | 17 | 16, 17 | 17.21 | 2.23 | 17 | 15, 20 | 17.87 | 2.5 | 18 | 15, 18 | 16.74 | 2.44 | 17 | 16, 18 |
| BIS | 22.58 | 3.52 | 23 | 22, 24 | 22 | 5.2 | 22 | 16, 28 | 22.07 | 3.65 | 23 | 19, 24 | 21.21 | 4.51 | 22 | 20, 24 |
| Optim. | 11.08 | 4.9 | 11 | 10, 12 | 10.93 | 5.3 | 11.5 | 7, 17 | 11.93 | 4.61 | 11 | 9, 16 | 12.78 | 5.24 | 13 | 11, 15 |
| Anxi. | 28.14 | 8.15 | 29 | 26, 31 | 31 | 8.31 | 31 | 24, 39 | 28.67 | 8.1 | 28 | 23, 37 | 24.89 | 9.31 | 25 | 22, 28 |
| SP | 33.71 | 7.95 | 35 | 32, 37 | 31.5 | 10.92 | 31 | 22, 43 | 35.6 | 9.01 | 37 | 29, 40 | 31.36 | 10.43 | 33 | 30, 37 |
| SR | 23.3 | 6.67 | 23 | 21, 25 | 22.43 | 7.14 | 23.5 | 13, 29 | 24.44 | 6.24 | 26 | 18, 29 | 26.93 | 7.22 | 27 | 24, 28 |
| J. Bias | -0.43 | 0.54 | -0.5 | -0.8, -0.2 | -0.38 | 0.56 | -0.55 | -1, 0.4 | -0.79 | 0.23 | -0.8 | -1, -0.6 | -0.4 | 0.52 | -0.4 | -0.6, -0.2 |
| BUT O | 0.9 | 0.64 | 0.9 | 0.67, 1 | 0.95 | 0.75 | 0.79 | 0.23, 1.7 | 0.85 | 0.45 | 1 | 0.45, 1.17 | 0.95 | 0.61 | 1 | 0.77, 1.1 |
| BUT P | 0.24 | 0.45 | 0.29 | 0.1, 0.4 | 0.19 | 0.29 | 0.25 | -0.03, 0.44 | 0.1 | 0.42 | 0.18 | -0.17, 0.43 | 0.2 | 0.6 | 0.13 | 0, 0.27 |
| Win | 11 | 10.83 | 8.28 | 5.9, 10.9 | 9.05 | 7.25 | 8.67 | 3.07, 14.29 | 12.43 | 13.35 | 7.39 | 2.9, 18.8 | 15.63 | 13.84 | 11.3 | 7.41, 14.3 |
| Lose | 49.97 | 13.58 | 50 | 57.9,54.4 | 55.59 | 12.16 | 52.94 | 45.7, 65.2 | 50.4 | 11.11 | 52.1 | 44.3, 59.2 | 55.58 | 12.73 | 55.03 | 51.7, 58 |
| Rever. | 8.96 | 4.38 | 8 | 7, 10 | 9 | 4.66 | 7 | 6, 15 | 9.6 | 5.25 | 9 | 5, 15 | 7.32 | 4.56 | 7.5 | 6, 8 |

|  | The Doubters | | | | The Knowers | | | | The Duffers | | | | The Consumers | | | |
| --- | --- | --- | --- | --- | --- | --- | --- | --- | --- | --- | --- | --- | --- | --- | --- | --- |
|  | M | SD | Mdn | Mdn 95% CI | M | SD | Mdn | Mdn 95% CI | M | SD | Mdn | Mdn 95% CI | M | SD | Mdn | Mdn 95% CI |
| Extra. | 3.1 | 1.64 | 3 | 2, 3.5 | 2.93 | 1.61 | 3 | 2, 3.5 | 2.67 | 1.3 | 2.5 | 2, 3.5 | 3.48 | 1.77 | 3.5 | 2.5, 4 |
| Consc. | 4.62 | 1.52 | 4.5 | 2, 5.5 | 4.78 | 1.71 | 5.25 | 3.5, 6 | 4.59 | 1.56 | 5 | 4, 6 | 5.39 | 1.44 | 6 | 5.5, 6 |
| Em. St. | 4.03 | 1.59 | 3.75 | 3.5, 4.5 | 4.09 | 1.43 | 4 | 3.5, 5 | 4.11 | 1.37 | 4 | 3.5, 4.5 | 4.54 | 1.65 | 4.5 | 4.5, 6 |
| Agree. | 4.3 | 1.17 | 4.5 | 4, 4.5 | 4.26 | 1.15 | 4 | 4, 4.5 | 4.57 | 1.09 | 4.5 | 4, 5 | 4.42 | 1.22 | 4 | 4, 4.5 |
| Open. | 4.98 | 1.28 | 5 | 4.5, 5.5 | 5.25 | 1.18 | 5.5 | 4.5, 6 | 4.93 | 1.43 | 5 | 4, 6 | 5.17 | 1.21 | 5 | 4.5, 5.5 |
| Author. | 16.48 | 6.71 | 17 | 15, 19 | 15.84 | 6.29 | 15.5 | 13, 20 | 15.29 | 6.55 | 14 | 12, 19 | 18.9 | 6.28 | 20 | 18, 22 |
| Super. | 11.36 | 4.12 | 11.5 | 10,13 | 11.55 | 4.25 | 12 | 11, 14 | 10.77 | 4.22 | 10 | 9, 12 | 13.09 | 4.47 | 14 | 12, 15 |
| Vanity | 17.78 | 5.47 | 18 | 15, 20 | 19 | 6.05 | 20 | 17, 20 | 17.14 | 5.66 | 18 | 16, 20 | 21.21 | 5.2 | 21 | 19, 24 |
| Entitl. | 10.09 | 3.35 | 10 | 9, 11 | 10 | 3.53 | 9 | 8, 12 | 10.11 | 3.53 | 9 | 8, 12 | 12.6 | 4.52 | 13 | 11, 14 |
| Self S. | 21.57 | 4.65 | 22 | 20, 24 | 21.47 | 3.99 | 21.5 | 20, 23 | 20.34 | 3.75 | 20 | 19, 21 | 22.6 | 3.53 | 23 | 21, 24 |
| Exploit. | 10.45 | 4.91 | 10 | 8, 12 | 9.42 | 4.12 | 8 | 7, 10 | 10.77 | 4.97 | 10 | 7, 13 | 11.23 | 4.94 | 10 | 9, 13 |
| Exhib. | 12.38 | 4.97 | 12 | 10, 14 | 11.53 | 4.64 | 12 | 9, 14 | 11.97 | 4.66 | 12 | 9, 15 | 13.02 | 4.89 | 13 | 10, 15 |
| Narcis. | 100.1 | 21.49 | 102.5 | 92, 109 | 98.82 | 20.67 | 83.25 | 94, 108 | 96.4 | 24.56 | 97 | 84, 107 | 112.6 | 22.12 | 114 | 105, 124 |
| BAS D. | 9.62 | 2.27 | 9.5 | 9, 10 | 9.81 | 2.5 | 10 | 9, 11 | 9.74 | 2.63 | 9 | 9, 10 | 11.06 | 2.32 | 11 | 10, 12 |
| BAS F. | 11.4 | 2.18 | 11 | 11, 12 | 11.37 | 2.46 | 11 | 10, 13 | 10.63 | 2.6 | 11 | 10, 12 | 12.13 | 2.03 | 12 | 11, 13 |
| BAS R. | 16.36 | 2.56 | 17 | 16, 18 | 16.42 | 2.81 | 17 | 15, 18 | 15.69 | 2.47 | 15 | 15, 17 | 17.49 | 2.09 | 18 | 16, 19 |
| BIS | 22.38 | 4.06 | 23 | 22, 25 | 22.79 | 4.03 | 23 | 22, 25 | 21.14 | 4.07 | 21 | 20, 24 | 21.34 | 4.2 | 21 | 20, 24 |
| Optim. | 11.47 | 5.59 | 11 | 9, 14 | 11.5 | 4.76 | 12 | 9, 14 | 11.14 | 3.82 | 11 | 10, 13 | 12.94 | 5.38 | 14 | 11, 16 |
| Anxi. | 27.55 | 9.73 | 29 | 24, 32 | 29.63 | 7.02 | 31 | 27, 33 | 25.8 | 7.55 | 26 | 22, 28 | 25.51 | 9.39 | 24 | 21, 31 |
| SP | 33.19 | 10.06 | 33.5 | 31, 38 | 34.24 | 8.7 | 36 | 31, 39 | 32.97 | 8.29 | 34 | 31, 37 | 30.96 | 9.77 | 32 | 25, 36 |
| SR | 24.57 | 6.79 | 23.5 | 21, 27 | 23.16 | 6.76 | 23 | 19, 25 | 24.6 | 8.23 | 25 | 19, 28 | 26.43 | 6.68 | 26 | 24, 29 |
| J. Bias | -0.43 | 0.53 | -0.6 | -0.8, -0.3 | -0.49 | 0.53 | -0.7 | -1, -0.2 | -0.39 | 0.53 | -0.5 | -0.7, -0.2 | -0.41 | 0.55 | -0.5 | -0.8, -0.3 |
| BUT O | 0.97 | 0.59 | 1 | 0.8, 1.12 | 0.96 | 0.11 | 0.96 | 0.7, 1.2 | 0.94 | 0.64 | 0.89 | 0.5, 1.1 | 0.86 | 0.54 | 0.84 | 0.6, 1 |
| BUT P | 0.22 | 0.48 | 0.27 | 0.05, 0.5 | 0.11 | 0.57 | 0.15 | 0, 0.39 | 0.21 | 0.59 | 0.3 | 0, 0.4 | 0.2 | 0.51 | 0.17 | 0, 0.24 |
| Win | 12.9 | 10.83 | 10.35 | 7.4, 14.1 | 10.1 | 9.98 | 7.34 | 4.6,10.5 | 16.9 | 16 | 11.2 | 6.9, 17.6 | 12.1 | 12.24 | 8.9 | 5, 11.6 |
| Lose | 50.8 | 14.22 | 53.64 | 49, 56 | 51.53 | 13.55 | 51.79 | 51, 60 | 53.82 | 9.21 | 53.85 | 51, 60 | 55.09 | 13.72 | 52.94 | 49, 61.5 |
| Rever. | 8.38 | 4.44 | 7.5 | 6, 9 | 8.84 | 4.4 | 8 | 7, 10 | 7.49 | 5.25 | 7 | 4, 9 | 8.49 | 4.48 | 8 | 6, 10 |

Extra. – extraversion, Consc. – conscientiousness, Em. St. – emotional stability, Agree. – agreeableness, Open. – openness to experience, Author. – authority, Super. – superiority, Entitl. – entitlement, Self S. – self-sufficiency, Exploit. – exploitativeness, Exhib. – exhibitionism, Narcis. – grandiose narcissism, BAS D. – BAS drive, BAS F. – BAS fun seeking, BAS R. – BAS reward responsiveness, Optim. – optimism, Anxi – anxiety, SP – sensitivity to punishment, SR – sensitivity to reward, J. Bias – judgment bias, BUT O – optimistic belief updating, BUT P – pessimistic belief updating, Win – win-shift, lose – lose-shift, Rever. – reversals
